# Early Mesozoic Coexistence of Amniotes and Hepadnaviridae

**DOI:** 10.1371/journal.pgen.1004559

**Published:** 2014-12-11

**Authors:** Alexander Suh, Claudia C. Weber, Christian Kehlmaier, Edward L. Braun, Richard E. Green, Uwe Fritz, David A. Ray, Hans Ellegren

**Affiliations:** 1 Department of Evolutionary Biology (EBC), Uppsala University, Uppsala, Sweden; 2 Museum of Zoology, Senckenberg Research Institute and Natural History Museum, Dresden, Germany; 3 Department of Biology and Genetics Institute, University of Florida, Gainesville, Florida, United States of America; 4 Department of Biomolecular Engineering, University of California, Santa Cruz, Santa Cruz, California, United States of America; 5 Department of Biochemistry, Molecular Biology, Entomology and Plant Pathology, Mississippi State University, Mississippi State, Mississippi, United States of America; 6 Institute for Genomics, Biocomputing and Biotechnology, Mississippi State University, Mississippi State, Mississippi, United States of America; University of Utah School of Medicine, United States of America

## Abstract

Hepadnaviridae are double-stranded DNA viruses that infect some species of birds and mammals. This includes humans, where hepatitis B viruses (HBVs) are prevalent pathogens in considerable parts of the global population. Recently, endogenized sequences of HBVs (eHBVs) have been discovered in bird genomes where they constitute direct evidence for the coexistence of these viruses and their hosts from the late Mesozoic until present. Nevertheless, virtually nothing is known about the ancient host range of this virus family in other animals. Here we report the first eHBVs from crocodilian, snake, and turtle genomes, including a turtle eHBV that endogenized >207 million years ago. This genomic “fossil” is >125 million years older than the oldest avian eHBV and provides the first direct evidence that Hepadnaviridae already existed during the Early Mesozoic. This implies that the Mesozoic fossil record of HBV infection spans three of the five major groups of land vertebrates, namely birds, crocodilians, and turtles. We show that the deep phylogenetic relationships of HBVs are largely congruent with the deep phylogeny of their amniote hosts, which suggests an ancient amniote–HBV coexistence and codivergence, at least since the Early Mesozoic. Notably, the organization of overlapping genes as well as the structure of elements involved in viral replication has remained highly conserved among HBVs along that time span, except for the presence of the *X* gene. We provide multiple lines of evidence that the tumor-promoting X protein of mammalian HBVs lacks a homolog in all other hepadnaviruses and propose a novel scenario for the emergence of *X* via segmental duplication and overprinting of pre-existing reading frames in the ancestor of mammalian HBVs. Our study reveals an unforeseen host range of prehistoric HBVs and provides novel insights into the genome evolution of hepadnaviruses throughout their long-lasting association with amniote hosts.

## Author Summary

Viruses are not known to leave physical fossil traces, which makes our understanding of their evolutionary prehistory crucially dependent on the detection of endogenous viruses. Ancient endogenous viruses, also known as paleoviruses, are relics of viral genomes or fragments thereof that once infiltrated their host's germline and then remained as molecular “fossils” within the host genome. The massive genome sequencing of recent years has unearthed vast numbers of paleoviruses from various animal genomes, including the first endogenous hepatitis B viruses (eHBVs) in bird genomes. We screened genomes of land vertebrates (amniotes) for the presence of paleoviruses and identified ancient eHBVs in the recently sequenced genomes of crocodilians, snakes, and turtles. We report an eHBV that is >207 million years old, making it the oldest endogenous virus currently known. Furthermore, our results provide direct evidence that the Hepadnaviridae virus family infected birds, crocodilians and turtles during the Mesozoic Era, and suggest a long-lasting coexistence of these viruses and their amniote hosts at least since the Early Mesozoic. We challenge previous views on the origin of the oncogenic *X* gene and provide an evolutionary explanation as to why only mammalian hepatitis B infection leads to hepatocellular carcinoma.

## Introduction

Viruses and their hosts share a rich coevolutionary past that is evidenced by a plethora of viral relics buried within host genomes. A striking example for this is the human genome where genomic relics of ancient, endogenized viruses constitute ∼8% of its total sequence [1]. These “fossils” of viruses have been collectively termed endogenous viral elements (EVEs) [2] and originate from host germline integration, followed by vertical transmission and subsequent fixation of virus-derived DNA in the genome of the host population [3], [4], [5]. The recent and ongoing availability of numerous genome sequences from non-model organisms [6], [7] has given rise to the field of paleovirology [8], the study of anciently integrated viruses, and has yielded the first direct insights into the long-term evolution of certain virus families [2], [9], [10]. The vast majority of EVE copies belongs to the Retroviridae family [1], [5] of viruses which rely on reverse transcription and obligate host genome integration, however, paleovirology has unearthed genomic fossils of all other major groups of eukaryotic viruses [2], [5], [11]. Whenever an EVE is present at a unique genomic location, it is possible to date the upper and lower age boundary of viral endogenization events by comparison of orthologous EVE insertions among different host species [5], providing direct evidence for host-virus coexistence.

The Hepadnaviridae are a family of reverse-transcribing dsDNA viruses infecting various species of birds [12] and mammals, including bats [13], rodents [14], and primates [15]. In humans, hepatitis B virus (HBV) poses one of the most widespread global health problems that affects more than 2 billion people and leads to >500,000 deaths per year [16]. Despite the availability of a number of primate genome sequences [7], HBV EVEs are absent or undetectable in these and other mammalian genomes [10]. In contrast, many bird genomes contain HBV fossils, such as the zebra finch and other songbirds [2], [10], [17], the budgerigar [18], [19], and other representatives of Neoaves [10]. Direct evidence from paleovirology suggests a coexistence of birds and HBVs that spans ∼70 million years (MY) of the Mesozoic and Cenozoic Eras, with HBV endogenizations dating from >82 million years ago (MYA) to <12.1 MYA [10]. Based on this fossil record, Hepadnavirus evolution might have either been characterized by an ancient coexistence with amniotes [10] or by a more recent bird-mammal host switch [10], the latter being in line with the paucity of extant host species and lack of mammalian HBV EVEs. The validity of either hypothesis is largely dependent on the genomic fossil record of HBVs [10]. The same is also the case for the enigmatic origin of the *X* gene of mammalian HBVs, as *X* appears to be absent in ancient avian HBV EVEs [10], while some extant avian HBVs exhibit an *X*-like gene [20]. The *X* gene is known to be involved in the generation of liver tumors in chronic HBV infection in humans and woodchucks [21], [22], [23], [24], [25], [26], so the elucidation of the evolutionary emergence of the *X* gene is of broad relevance to biological and medical research on hepatitis B viruses.

Here we report endogenous hepadnaviruses from recently sequenced turtle [27], [28], snake [29], and crocodilian genomes [30], [31]. Among these EVEs is a near-complete crocodilian HBV genome from the Late Mesozoic and an Early Mesozoic turtle HBV, providing us with the unprecedented opportunity to study the host range, genome evolution and deep phylogeny of Hepadnaviridae. We show that genome organization and replication is highly conserved among HBVs with the exception of the presence of the oncogenic *X* gene, for which we infer an evolutionary scenario of *de-novo* emergence in the ancestor of mammalian HBVs. Finally, our hepadnaviral fossil record reveals Mesozoic coexistence of Hepadnaviridae with three of their five major host taxa and supports a scenario of ancient amniote–HBV cospeciation.

## Results

### Evidence for Endogenous Hepadnaviruses (eHBVs) in Crocodilian, Snake, and Turtle Genomes

**Figure 1:**
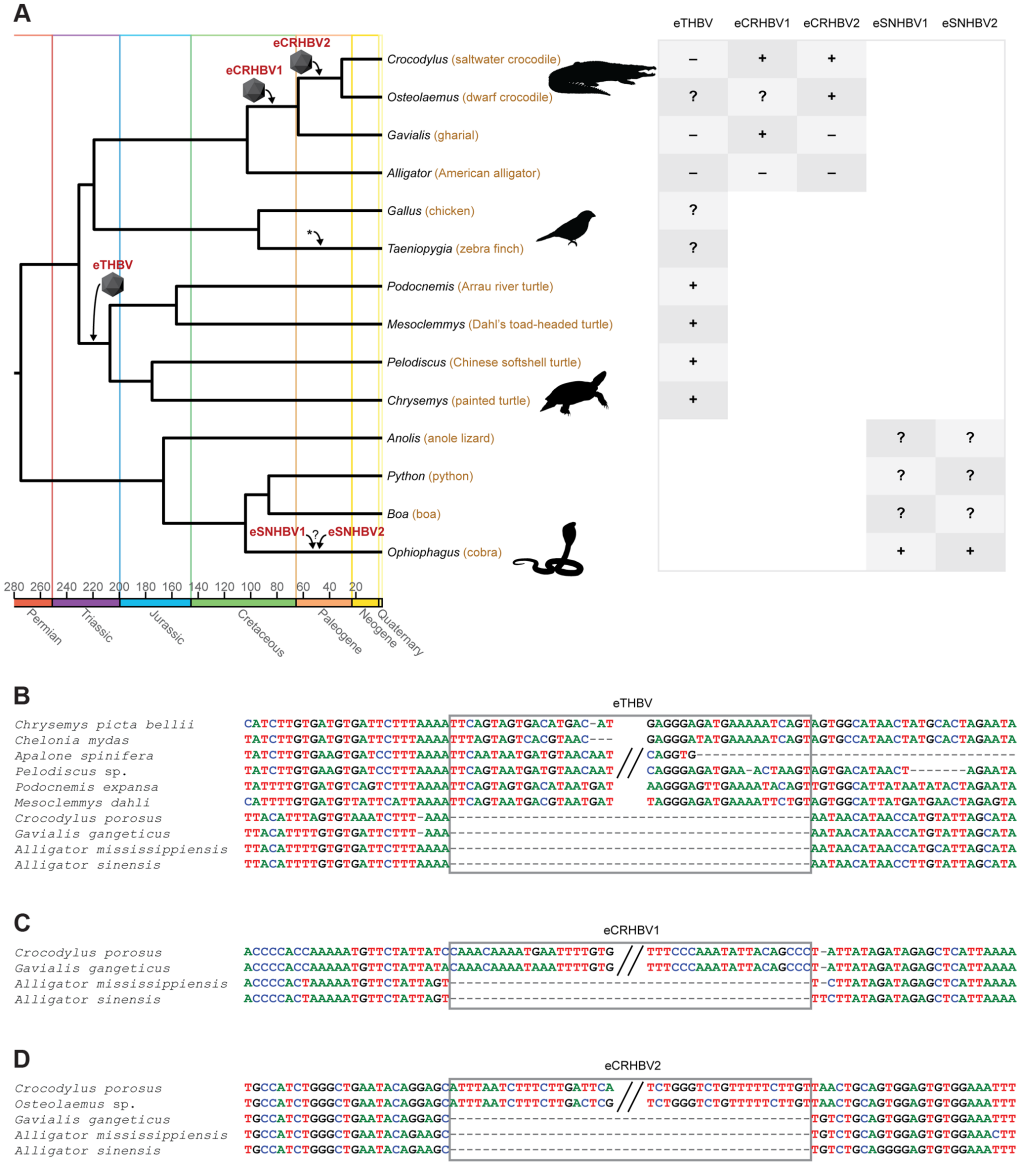
Non-avian hepatitis B paleovirus endogenization events. (A) Simplified chronogram of non-mammalian amniotes based on molecular dates of phylogenetic relationships among amniotes [40], squamate lepidosaurs [78], [79], turtles [39], birds [80], and crocodilians [42], [43]. Icosahedrons denote endogenization events, the asterisk indicates previously studied avian endogenizations [10], and the colored time axis corresponds to the International Stratigraphic Chart (http://www.stratigraphy.org/ICSchart/StratChart2010.pdf). All HBV EVE endogenization events were reconstructed based on their respective presence/absence patterns (“+”: presence, “−” absence; “?”: missing data or sequence could not be aligned). This is with the exception of cobra eSNHBVs where we could not ascertain presence/absence states in other squamates. The early Mesozoic eTHBV paleovirus (B) is present in orthologous locations in both pleurodiran and cryptodiran turtles, but absent in crocodilians. All crocodilians to the exclusion of the alligator (i.e., Longirostres [41]) share the Cretaceous eCRHBV1 insertion (C), while the Paleogene eCRHBV2 insertion (D) is present in saltwater and dwarf crocodile (i.e., Crocodylidae), but absent in orthologous positions in gharial and alligator. HBV-derived sequence residues are boxed.

We searched the recent saltwater crocodile, gharial, and American alligator draft genome assemblies [30], [32] using whole viral genomes of the duck HBV (DHBV; AY494851) and the Mesozoic avian eHBV (eZHBV_C [10]), and identified two endogenous crocodilian HBVs (eCRHBVs; Fig. 1A). Likewise, we screened the genomes of turtles (painted turtle, softshell turtles, and sea turtle [27], [28]), squamate lepidosaurs (cobra, boa, python, and anole lizard [29], [33], [34], [35]), and mammals (human, opossum, and platypus [36], [37], [38]) for the presence of eHBVs. We detected a single locus in turtle genomes, hereafter referred to as endogenous turtle HBV (eTHBV; Fig. 1A), two endogenous snake HBVs in the cobra genome (eSNHBVs; Fig. 1A), but no EVEs in the remaining squamate and mammalian genomes. Our presence/absence analyses show that all four available cryptodiran turtle genomes plus the sampled pleurodiran (side-necked) turtles (*Mesoclemmys* and *Podocnemis*) exhibit the eTHBV insertion, while it is absent in the orthologous position in crocodilian genomes (Fig. 1B). This suggests that it is of Triassic origin and was endogenized in the ancestor of Testudines that lived 207.0–230.7 MYA [39], [40]. eCRHBV1 (Fig. 1C) is present in all crocodilians except alligators (i.e., Longirostres [41]) and is 63.8–102.6 MY [42] old, i.e., of Cretaceous origin. The second crocodilian EVE (eCRHBV2) is exclusively shared between saltwater and dwarf crocodile; its endogenization thus occurred during the Paleogene in the ancestor of Crocodylidae (30.7–63.8 MYA [42], ). Unfortunately, the snake EVEs remain undated, as none of the cobra eSNHBV loci could be aligned to other squamate genomes for ascertainment of EVE presence/absence (Fig. 1A). Given the dense fossil record of crocodilians and turtles that provides multiple calibrations for molecular dating of species divergences [39], [42], we suggest that the aforementioned dates are robust age estimates of eCRHBV1, eCRHBV2, and eTHBV endogenizations. Furthermore, molecular dating studies using mitochondrial genomes [44], [45] or nuclear loci [42], [43] yielded similar results on crocodilian divergence times, and the basal turtle divergence time of 207 MYA [39], [46] (i.e., the Cryptodira–Pleurodira split) is a nuclear estimate that is well compatible with mitochondrial estimates [47], [48] and the fossil record [49].

Annotation assigns these five eHBV insertion sequences no extant protein-coding function in their hosts' genomes (see GenomeBrowser [50]; the two crocodilian eHBVs are located within very large introns and the snake eHBV loci are undetectable in the lizard genome, while the turtle eHBV appears to constitute intergenic sequence). In line with this, we identified several frameshifting indels and premature stop codons in all five eHBVs (S1 Table). Most of these were lineage-specific and found at different locations, indicating that they were not present in the common ancestor where the viral integration occurred. To determine whether any of the eHBV fragments may still show any sign of having been functional in the past before incurring stops and frameshifts, we performed likelihood ratio tests of the ratio of the rate of non-synonymous to the rate of synonymous substitutions (ω) either fixed to 1 or being allowed to vary freely. As none of these tests provided statistical support for deviation from ω = 1 (S2 Table), there was thus no evidence for non-neutral evolution of these loci in the sampled genomes. Similar observations were previously made in selection tests on avian eHBVs where neutrality could not be rejected [17], which may suggest that none of the currently known HBV EVEs possess an obvious protein-coding function in their host genomes. The crocodilian and turtle eHBVs' GC content is similar to the GC level of the adjacent flanking sequence of the host (S1 Figure), which suggests that they have resided in the host genome for long enough to show a host-like base composition.

Given that we detected no sign of non-neutral evolution of the crocodilian and turtle eHBV loci since their respective endogenization events, another line of evidence for the antiquity of their integration is the level of sequence divergence between orthologous eHBVs. We therefore calculated distances per eHBV locus (see Materials and Methods) and applied neutral substitution rates for crocodilians (3.9×10^−10^ substitutions/site/year [30]) and turtles (8.43×10^−10^ substitutions/site/year for *Pelodiscus* sp. and 4.77×10^−10^ substitutions/site/year for *Chelonia mydas* [30]) to determine locus-specific estimates of respective endogenization times. Consequently, we inferred integration events to have happened 70.3 MYA in eCRHBV1, 20.5 MYA in eCRHBV2, and 179.0 or 316.3 MYA in eTHBV. While these dates are compatible with our lower age boundaries of endogenization events derived from eHBV presence/absence patterns (Fig. 1A), we suggest that the distance-based values are less robust estimates, as they rely on a limited number of nucleotides from a single genomic locus and are thus easily prone to biases caused by, for example, variation in substitution rates among lineages (e.g., *Pelodiscus* vs. *Chelonia*) or among genomic regions.

### Conserved Genome Organization and Structural Features of Crocodilian, Snake, and Turtle eHBVs

**Figure 2:**
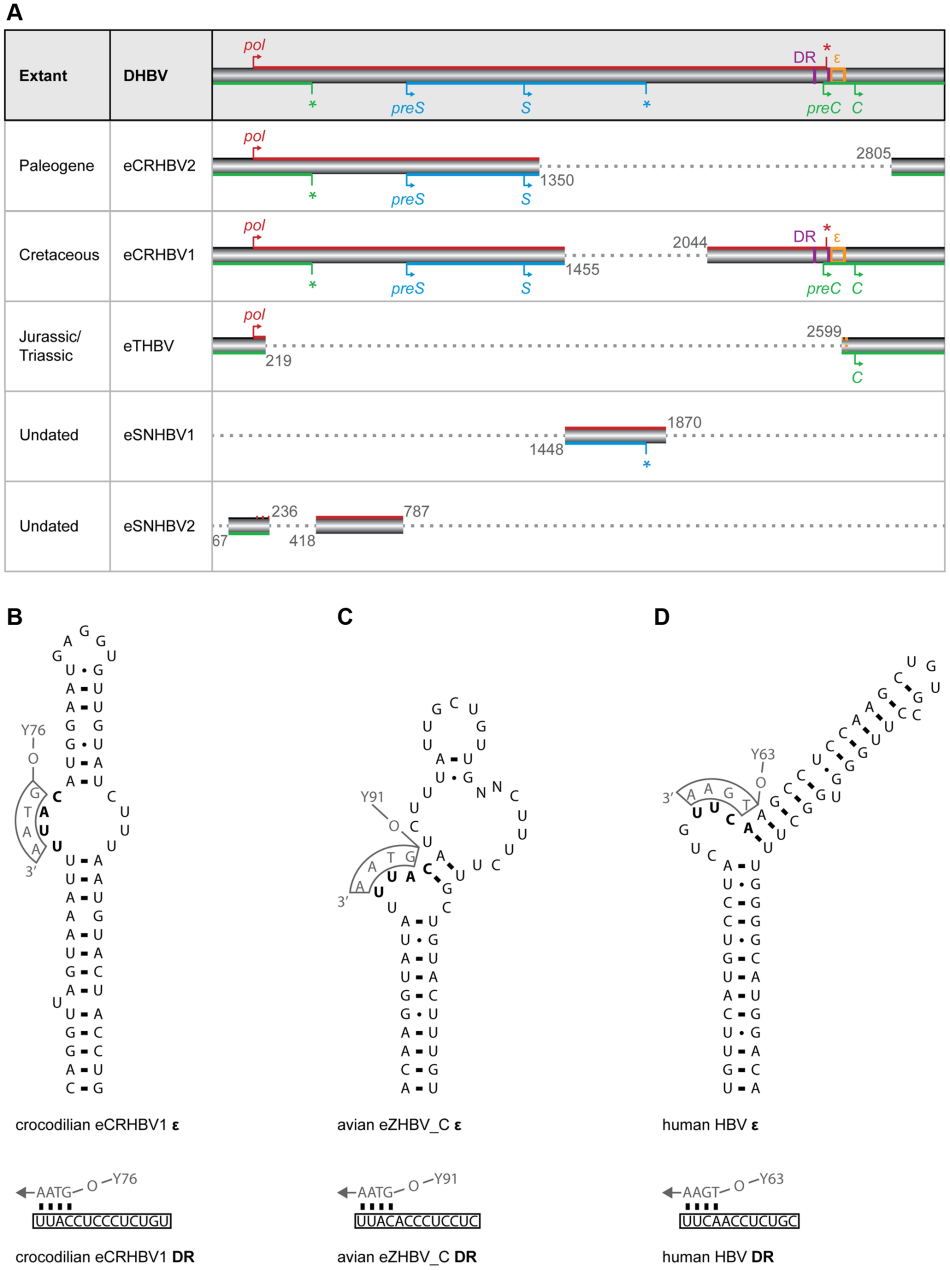
Non-avian hepatitis B paleovirus genome organization and features of viral replication. (A) The fragments of the crocodilian and turtle HBV EVEs described herein extend over ∼81% (eCRHBV1), ∼52% (eCRHBV2), ∼21% (eTHBV), ∼18% (eSNHBV2), and ∼14% (eSNHBV1) of the circular 3,024-bp extant DHBV genome from ducks (AY494851). Genomic regions missing from these fragments are indicated by dashed grey lines. The reconstructed start (arrow) and stop (asterisk) codon positions for the ORFs of the polymerase protein (*pol*; red), the presurface/surface protein (*preS/S*; blue), and the precore/core protein (*preC/C*; green) indicate a highly similar genome organization among Hepadnaviridae. Genomic features related to viral replication are direct repeats (DR; purple vertical lines) and the RNA encapsidation signal (ε; orange box); these are contained in the crocodilian eCRHBV1 fragment. Comparison of DR sequence and ε RNA secondary structure of this crocodilian HBV EVE (B) with homologous structures in the Mesozoic avian HBV EVE (C) and human HBV [51] (D) shows conservation of the priming bulge (bold), whereas the rest of the stably base-pared ε hairpin structure exhibits little sequence similarity [51] between avian and mammalian HBVs, and also the crocodilian HBV (see also S1 Figure for an alignment of ε sequences). In extant HBVs [51], the conserved tyrosine (Y) residue of the terminal protein domain (numbers indicate the tyrosine amino acid site) of the *pol* ORF is attached to a DNA primer (grey letters) that binds to the ε priming bulge and the 5′ end of the DR. Arrows depict the direction of minus DNA synthesis.

Extant avihepadnaviruses (avian HBVs) and orthohepadnaviruses (mammalian HBVs) have a circular genome organization with overlapping open reading frames (ORFs) and a streamlined genome size of about 3.0 kb and 3.2 kb, respectively [14]. The crocodilian, snake, and turtle eHBV fragments comprise up to 81% of an *Avihepadnavirus* genome (Fig. 2A), permitting us to reconstruct large portions of their genome organization. We detected overlapping regions of the precore/core (*preC/C*) ORF with the polymerase (*pol*) ORF (eCRHBVs and eTHBV; Fig. 2A) and of the presurface/surface (*preS/S*) ORF with the *pol* ORF (eCRHBVs and eSNHBV1; Fig. 2A), which suggests that all known extant and fossil avian, crocodilian, and mammalian HBVs exhibit a highly similar genome organization. This probably also applies to snake and turtle HBVs, because, while the eSNHBV1 and eTHBV fragments only span ∼14 and ∼21% of an HBV genome, they contain a region of overlapping ORFs (Fig. 2A). Finally, we used approaches based on similarity searches and alignments, and did not detect any evidence for an *X* ORF in our non-avian eHBVs (Fig. 2A).

In addition to protein-coding sequences, we detected genomic features related to viral replication (Fig. 2B–D), as the near-complete eCRHBV1 genome comprises the region where avihepadnaviruses and orthohepadnaviruses contain direct repeats (DR) and the RNA encapsidation signal (ε). This region lies within the end of the *pol* ORF and the start of the *preC/C* ORF [51] (Fig. 2A), but eCRHBV1 exhibited no significant nucleotide sequence similarity against DR+ε sequences of avian and mammalian HBVs. Yet, our structural analyses identified a DR motif of 14 nt that is present in identical copies within *pol* (DR2) and *preC/C* (DR1). We further detected a 54-nt RNA hairpin motif with a priming bulge (5′–UUAC–3′) identical to the first four RNA nucleotides of the DR motif and reverse complementary to the (–)-DNA primer in avian HBVs [51], suggesting that this is a structure that functionally corresponds to ε of extant HBVs (Fig. 2B). In avian and mammalian HBVs, ε interacts with the (–)-DNA primer that is covalently linked to the conserved tyrosine residue of the terminal protein (TP) domain of the Pol protein [51] and establishes encapsidation of viral pregenomic RNA [52], [53] as well as reverse transcription into viral (–)-DNA [53], [54]. Despite the lack of sequence similarity between avian and mammalian HBV ε [51], as well as the putative crocodilian HBV ε (see S2 Figure), hepadnaviral replication appears to require strong structural constraint on ε with regards to stable base-pairing, as well as the presence of a bulge region and an apical loop (Fig. 2B–D and refs. [19], [51], [55]). Only the 4-nt binding sites for the (–)-DNA primer within DR and ε exhibit sequence conservation among Hepadnaviridae (Fig. 2B–D and S2 Figure).

### Phylogenetic Relationships of Crocodilian, Snake, and Turtle eHBVs within Hepadnaviridae

**Figure 3:**
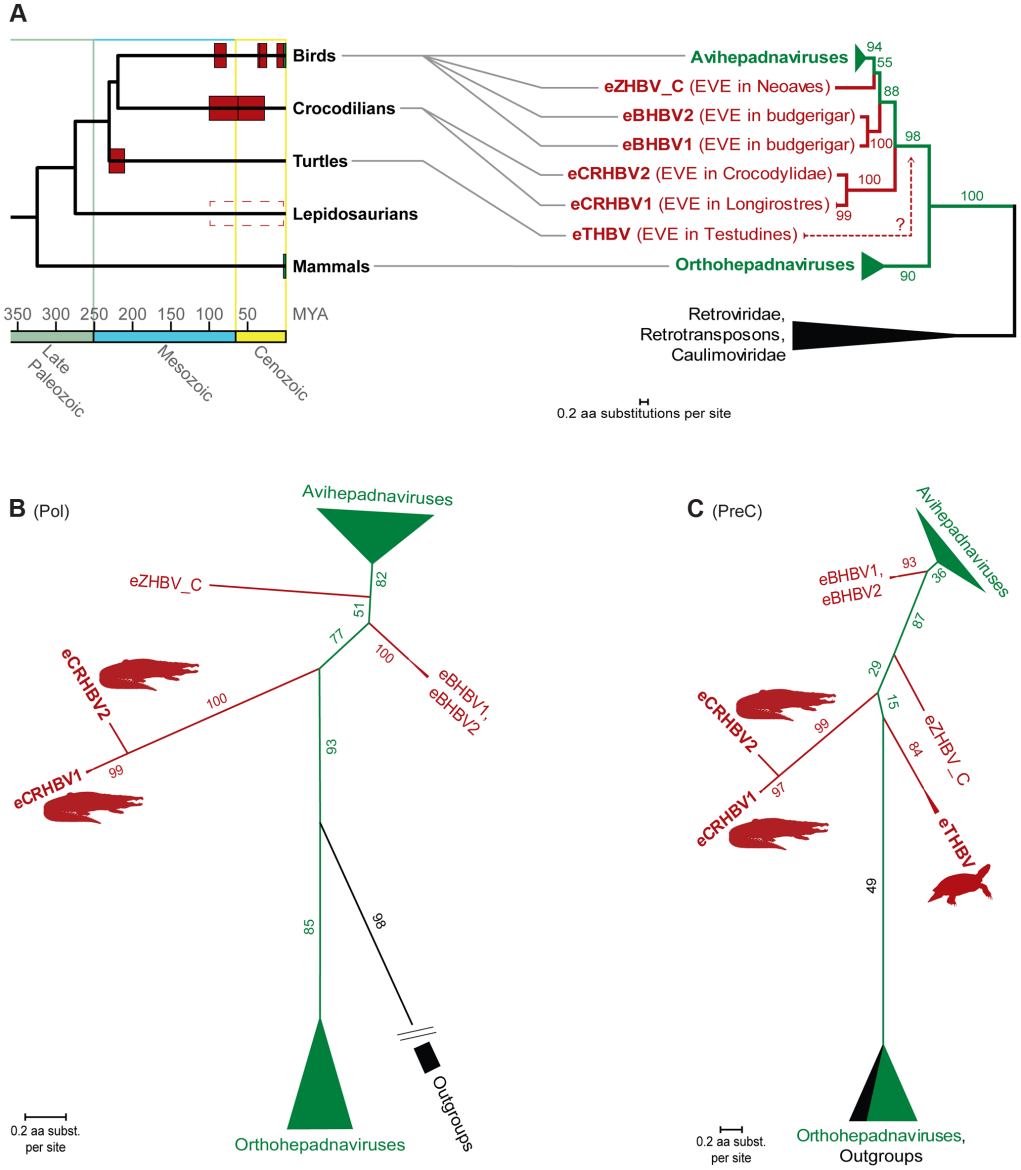
Phylogeny of Hepadnaviridae and their amniote hosts. (A) Plotting the HBV endogenization events (red boxes) reconstructed in this study and ref. [10] on a dated [40] consensus phylogeny of amniotes [27], [30] suggests temporary Mesozoic coexistence of Hepadnaviridae with birds, crocodilians, and turtles, respectively. Extant coexistence with birds and mammals is denoted by green boxes and the undated evidence for snake HBV endogenization events is indicated by a dashed box. The rooted (A) and unrooted (B) phylograms (see S4 Figure for phylograms including the short fragments of eSNHBV1 and eSNHBV2) of a maximum likelihood (ML) analysis of the polymerase protein from hepadnaviruses and reverse-transcribing outgroups (caulimoviruses, retroviruses and retrotransposons) exhibit a phylogenetic placement of crocodilian eHBVs that recapitulates the deep phylogeny of their amniote hosts. The precore/core protein ML phylogram (C) on the same ingroup sampling (plus eTHBV) topologically indicates a bird+crocodilian grouping, too, as well as an affinity of these HBVs to the turtle eHBV. Nevertheless, the resolution of the PreC protein on deep HBV relationships remains limited as suggested by low bootstrap support of some internodes and different topologies with regards to the outgroup (i.e., grouping them within Orthohepadnaviruses) as well as the branching order of avian eHBVs. ML bootstrap values are shown in % on respective nodes. Note the long internodes leading to non-avian eHBV branches; these further support the distinctiveness of the protein sequences of these ancient hepadnaviral lineages. All ML trees were generated via RAxML 7.4.7 [76] using the JTT+G model and 1000 bootstrap replicates.

Recent paleovirological studies on avian eHBVs suggest that extant avihepadnaviruses and orthohepadnaviruses exhibit relatively shallow branches within phylogenetic trees compared to the deep divergences among eHBVs [5], [10] (see also S3C Figure). This suggests a recent divergence of circulating viruses among each of these two HBV lineages, whereas their endogenous avian counterparts appear to be relics of several distantly related, ancient lineages [10], [18] with avihepadnaviruses being sister clade to one of them [10]. We reevaluated this by inferring the phylogenetic relationships based on Pol and PreC/C protein sequences of the non-avian eHBVs among Hepadnaviridae. In addition to full-length avian eHBVs and a dense sampling of extant HBVs, we included reverse-transcribing outgroups such as retroviruses, caulimoviruses, and retrotransposons. In phylogenetic trees of both Pol and PreC/C (Fig. 3B–C, S4A–C Figure), the avian eHBVs form ancient, unrelated lineages, but with an eZHBV_C+avihepadnaviruses clade in the Pol tree and an eBHBVs+avihepadnaviruses clade in the PreC/C tree. This reversal in branching order could be explained by interspecific viral recombination events, as have been observed in some extant HBV lineages [56], [57], but is more likely due to the very limited amount of phylogenetically informative characters in the short PreC/C protein.

Irrespective of the branching order of avian eHBVs, the two crocodilian eHBVs (eCRHBV1 and eCRHBV2) consistently group together as a third major hepadnaviral lineage, and form the sister group of all avian HBVs and eHBVs, which is supported with high bootstrap values in the Pol tree (Fig. 3B). This grouping, of course, is largely dependent on the position of the root of the Hepadnaviridae phylogeny. Our dense ingroup and outgroup sampling yields a Pol tree topology that strongly suggests Orthohepadnaviridae as the first branch among HBVs with respect to the remaining hepadnaviral lineages. Thus, in relation to avian and mammalian HBVs, the phylogenetic position of crocodilian HBVs reflects the host phylogenetic relationships between birds, crocodilians, and mammals [27], [30], [40] (Fig. 3A). Unfortunately, it is not possible to include eTHBV in this well-resolved Pol tree, as the turtle EVE spans only a small part (16 aa) of the Pol sequence. Consequently, the phylogenetic affinities of eTHBV are solely inferred from the PreC/C tree, which exhibits a lack of bootstrap support on its backbone, presumably as a consequence of too few phylogenetically informative characters within the PreC/C protein (342 aligned aa sites). However, the PreC/C tree does recover eTHBV as sister lineage of crocodilian+bird HBVs, which supports the above-mentioned similarity of the deep phylogenetic relationships among HBVs, as well as among their amniote hosts [27], [30], [40] (Fig. 3A). Finally, with regards to snake eHBV affinities, the short sequences of eSNHBV1 (141 aa Pol) and eSHBV2 (57 aa PreC and 123 aa Pol) hamper a well-supported resolution of the tree backbones, yet there is topological indication for a grouping of eSNHBV1 with avian HBVs+eHBVs (S4A Figure) and eSNHBV2 with crocodilian eHBVs (S4B–C Figure).

### *De-novo* Emergence of the Oncogenic *X* Gene in Orthohepadnaviruses

**Figure 4:**
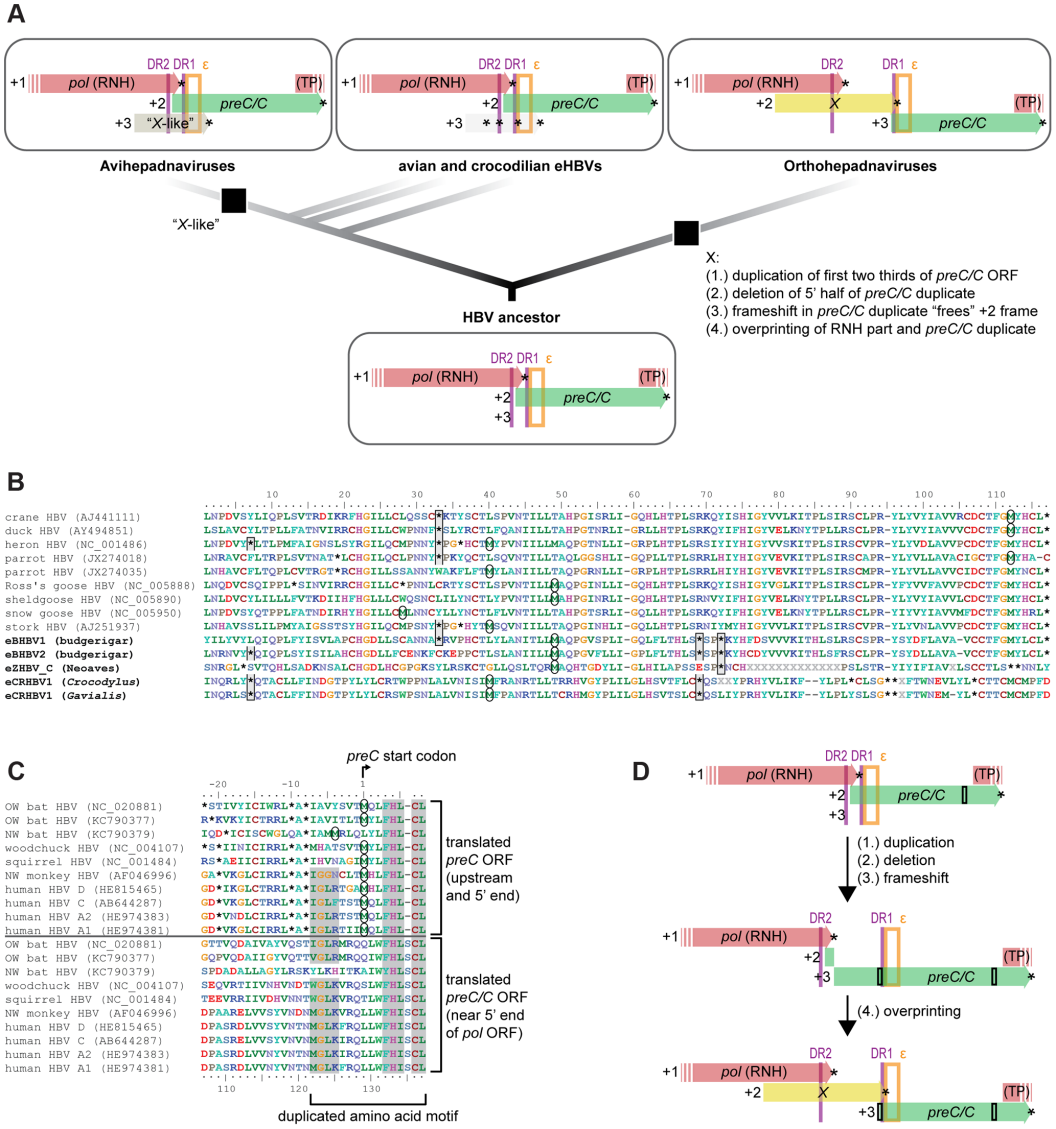
An evolutionary scenario for the emergence of the oncogenic *X* gene. (A) The genome of the HBV ancestor contained neither an *X* nor *X*-like ORF, given that avian and crocodilian eHBVs lack an *X*-like ORF, despite the fact that it is thought to be expressed in some closely related avihepadnaviruses [20]. *X* (+2 frame) and *X*-like (+3 frame) are encoded in different reading frames relative to the part of *pol* they overlap with (+1 frame), which strongly suggests that these ORFs are non-homologous and the *X*-like protein emerged in the ancestor of avihepadnaviruses. Independently, the *X* protein arose in orthohepadnaviruses via overprinting (4.) after a segmental duplication (1.), a partial deletion (2.), and a frameshifting mutation (3.) in one region of the HBV genome. Only the part of the HBV genome between the ribonuclease H (RNH) and the terminal protein (TP) domains of the *pol* ORF is shown, including structural elements such as direct repeats (DR; purple vertical lines) and the RNA encapsidation signal (ε; orange box). (B) Translated sequence alignment of the *X*-like ORF *sensu* Chang et al. [20] indicates presence of multiple internal stop codons in avian and crocodilian eHBVs, resulting in potential translation products <30 aa. Stop codon positions (asterisks) are highlighted with grey boxes if they are conserved between eHBVs, start codon positions for the longest possible ORF are highlighted by circles. Even when assuming that nonconventional start codons are used as suggested for DHBV [20], potential eHBV X-like proteins would comprise just a portion of the DHBV X-like protein. (C) Sequence similarity between translated *preC/C* 5′ end region (incl. in-frame aa sites upstream of the start codon) and translated central region of the *preC/C* ORF might be a potential remnant of an ancient segmental duplication of the first two thirds of the *preC/C* ORF. Amino acid residues with dark grey background are conserved between the start and the middle part of the *preC/C* ORF and thus constitute a potentially duplicated amino acid motif. (D) Schematic illustration of the proposed evolutionary steps of *X* ORF emergence [(1.) to (4.)] described in (A) that potentially led to the extant genome organization of orthohepadnaviruses. Black rectangles illustrate the location of the duplicated amino acid motif shown in (C).

Annotation suggests that an *X* or *X*-like ORF is absent in non-avian eHBVs, while the genomes of orthohepadnaviruses and avihepadnaviruses appear to contain an *X* and *X*-like gene, respectively (Fig. 4A). Even when aligning the translated sequences of eHBVs in the region homologous to the putative *X*-like ORF of avihepadnaviruses [20], all eHBVs and even several extant avian HBVs exhibit several internal stop codons at conserved positions (Fig. 4B), suggesting that an *X*-like ORF never existed in these unrelated HBV lineages. While it remains unclear whether the putative *X*-like gene in DHBV has a function [58], it is interesting to note that the ribonuclease H (RNH) domain (partially overlapping with the *X*/*X*-like ORF region) has a moderate GC content in eHBVs and avihepadnaviruses (S3 Figure), while mammalian HBV genomes exhibit a conserved *X* gene and a highly elevated GC content of the RNH domain.

Despite both overlapping with the RNH domain of the *pol* ORF, *X* and *X*-like ORFs are found in different reading frames (Fig. 4A). Considering that the Pol protein sequence is homologous among all HBVs and is encoded in the +1 frame, the fact that *X* resides in the +2 frame and *X*-like in the +3 frame counters homologization of the codon and protein sequence encoded in the *X* and *X*-like ORFs. This provides further evidence that the ancestor of Hepadnaviridae lacked an *X* or *X*-like gene and that the X protein arose *de novo* in the *Orthohepadnavirus* lineage [10]. The partially overlapping nature of *X* suggests that it emerged by using an unoccupied reading frame within a pre-existing ORF, a process termed overprinting [59]. We therefore conducted overprinting analyses (S3 Table) using the method described by Pavesi et al. [60] for detecting *de-novo* ORFs based on their codon usage. Although the *X* codon usage shows an expected weaker correlation with the rest of the viral genome than is the case with the other, older overlapping ORFs (S3 Table), subsampling analyses suggest that the overlapping part of the *X* ORF is too short to derive a statistically significant conclusion (S5 Figure).

In contrast to non-mammalian HBVs where the RNH domain of *pol* and the start of *preC/C* overlap, these two ORFs are instead disjoined from each other in mammalian HBVs and together encompass the non-overlapping part of the *X* ORF (Fig. 4A). It has been proposed that an ORF overlap can easily be eliminated in connection with a duplication of the particular region [61], which could well have been the case in the ancestor of orthohepadnaviruses and led to the present genome organization. This would also explain why, apart from the aforementioned differences, the locations of all other genomic features of this region have remained unchanged throughout HBV evolution, such as the exact location of DR1, DR2, and ε within the *pol* and *preC/C* ORFs. To test whether there are still detectable sequence remnants (i.e., duplicated amino acid motifs) of such an ancient segmental duplication, we screened the genomes of all orthohepadnaviruses against themselves as well as each other via translated nucleotide similarity searches and considered only hits that were in the same orientation in the HBV genome. Only one amino acid motif of considerable length (i.e., >9 translated aa on the same strand) appears to be duplicated (Fig. 4C) in the entire *Orthohepadnavirus* genome with up to 50% sequence similarity between the two copies. Both potential duplicates reside within the *preC/C* ORF, one of them at its very beginning and the other near the 5′ end of the *pol* ORF.

We therefore propose a novel scenario for *de-novo* emergence of the *X* ORF in orthohepadnaviruses (Fig. 4A). This builds on the suggestion by Pavesi et al. [60] that the overlapping part of *X* emerged *de novo* via overprinting of the *pol* RNH domain and is completed by our inference of the origin of the non-overlapping part of the *X* ORF. We hypothesize that the non-overlapping part of *X* arose by duplication of the first two thirds of the *preC/C* ORF that extended from the *preC/C* start to the above mentioned amino acid motif (Fig. 4D). A subsequent deletion of the first half of the first duplicate (Fig. 4D) purged the surplus in DR and ε motifs, potentially because it interfered with correct viral replication. If this coincided with the induction of a frameshift mutation (Fig. 4D) within the partial duplicate of *preC/C*, this shifted the intact downstream *preC/C* ORF (+2 frame) by one nucleotide (+3 frame) relative to *pol* that resides in the +1 frame. This would have thus prepositioned the +2 frame of the partial *preC/C* duplicate for overprinting (Fig. 4D), while keeping the intact *preC/C* ORF unaffected, as it resides in a different reading frame.

## Discussion

### Mesozoic Coexistence of Hepadnaviridae and Land Vertebrates

Our study, together with a previous study on a Mesozoic eHBV in birds [10], provides direct evidence for the coexistence of Hepadnaviridae and three of the five major clades of amniotes during the Mesozoic Era, two of which (i.e., crocodilians and turtles) were previously not thought to be candidate hosts of extant HBVs [14]. The latter is also the case for snakes. While the cobra eHBVs remain undated, the three datable non-avian eHBVs described herein are ≥30.7 MY old, so we assume that these non-avian EVEs constitute snapshots of an ancient but now extinct host-virus association. This is in line with the paucity of HBV endogenization events in crocodilian, snake, and turtle genomes, in contrast to birds where dozens of these occurred during their long-lasting and ongoing coexistence [2], . Furthermore, our non-avian HBV fossils suggest that the minimum age of definite existence of Hepadnaviridae is not >82 MY as suggested in ref. [10], but >207 MY and thus reaches far into the Mesozoic Era. When considering indirect paleovirological evidence such as our phylogenetic analysis grouping mammalian HBVs as sister to crocodilian+avian HBVs (but in disagreement with the apparent lack of mammalian HBV fossils [10]), Hepadnaviridae could be considered as a considerably older family of viruses with the root of all known HBVs at least in the Early Mesozoic or even in the common ancestor of Amniota.

### Lack of Hepadnaviral Fossils in Mammalian Genomes

The fact that we identified eHBVs in crocodilian, snake, and turtle genomes implies that Mammalia is the only major lineage of land vertebrates that lacks evidence for the existence of endogenous hepadnaviruses. Unfortunately, it was not possible to determine whether the cobra eSNHBVs or their flanking sequences are present or absent in other squamate lepidosaurs (anole lizard [35], python [34], and boa [33]), which can potentially be explained by the accelerated neutral substitution rate characteristic to this clade [27], [34] that, together with a very high rate of DNA loss [62], hampers the detection and comparison of orthologous non-functional genomic loci across this level of species divergence. Likewise, fast molecular evolution must have led to the scarcity of ancient transposable element (TE) insertions and retroviral EVEs in these genomes [62]. This is not expected in the case of the very slowly evolving genomes of turtles [27], [28] and crocodilians [30], [31], all of which are littered with ancient TEs [63], and readily explains why Mesozoic eHBVs are still detectable as such in their genomes, even after >200 MY of sequence decay and lack of selective constraint.

The absence of endogenous hepadnaviruses in mammals [10] despite dozens of available genome sequences [7] and a rich diversity of extant, exogenous HBV infections [13] remains puzzling. Under the scenario of an ancient coexistence/codivergence of amniotes and Hepadnaviridae, mammalian HBVs would have had equal time for recurring, stochastic germline endogenization of viral fragments as avian HBVs had since the speciation of their amniote ancestor. Also, relative to squamates, mammalian genomes appear to have a much slower rate of DNA loss [62] and a lower substitution rate [27], suggesting that a fixed HBV endogenization in the germline would have potentially been detectable even after many millions of years. Although the rate of mammalian sequence evolution is somewhat higher than that for birds [27], [64], it is less than that of squamates and therefore less likely to erase the evidence for a fixed HBV endogenization unless it were truly ancient. We conclude that, while the so far sequenced representatives of major mammalian lineages generally seem to lack eHBVs, it cannot be excluded that the foreseeable sequencing of thousands of additional mammalian genomes [6] might lead to the unearthing of recent, lineage-specific endogenizations of mammalian eHBVs.

### Genomic Stasis of Hepadnaviridae and *De-Novo* Origin of the X Protein

Given the evidence that hepadnaviruses coexisted with their amniote hosts at least since the Early Mesozoic, it is striking that the genome organization of HBVs have remained relatively stable over the course of >200 MY, including the patterns of overlapping protein-coding sequences and structures involved in viral replication. The only major difference among HBV genomes appears to be the presence or absence of an *X* gene. Our analyses provide multiple and independent lines of evidence that the common ancestor of Hepadnaviridae did not exhibit a fourth ORF and that the *X* gene therefore is an evolutionary novelty that arose in the *Orthohepadnavirus* lineage [10]. If the expressed X-like protein in duck HBVs is indeed functional [20], [65] (note that its function was questioned in ref. [58]), then this gene must have emerged *de novo* within avihepadnaviruses, as its putative ORF region is heavily disrupted by internal in-frame stop codons in all endogenous HBV lineages discovered so far. For example, in eCRHBV1 and eZHBV_C there are more premature stop codons in the ∼120 codons of the *X*-like ORF than in the total of >1100 codons of the three remaining ORFs together (compare Fig. 4B with S1 Table and ref. [10]). Most importantly, it has previously been overlooked that the *X* and *X*-like ORFs cannot represent a single, homologous origin of a gene by overprinting because they lie within different frames of the homologous region of the *pol* ORF that they overlap with. Any structural [66] or functional [20] similarities between the encoded proteins must have thus evolved independently. A scenario of *X* emergence via segmental duplication of *preC/C* and subsequent overprinting of parts of *pol* and *preC/C* ORFs provides a simple explanation to why the DR1 (nested at the 5′ end of *preC/C*) and DR2 (nested at the 3′ end of *pol*) sequences are separated by a few hundred bp of non-overlapping, *X*-specific sequence in orthohepadnaviruses, while they are only a few dozens of nucleotides apart from each other in non-mammalian HBVs where the *X* gene is missing and *pol*+*preC/C* are overlapping instead. It is worth noting that Liu et al. [19] recently reported an avian eHBV genome that was endogenized with partially duplicated *pol* and *preC/C* ORFs, suggesting that segmental duplications do occur during replication in the virus particle and also seem to be present in the viral DNA genome that resides in the host nucleus. Finally, the restriction of the presence of *X* to mammalian HBVs coincides with the notion that chronic HBV infection is associated with HCC development in mammals only, while avian HBVs do not seem to cause HCC in birds [20]. This further adds to the substantial evidence for an oncogenic effect of the *X* gene of orthohepadnaviruses [21], [22], [23], [24], [25], [26]. Although the X protein is known to have several indispensable functions in regulation of protein interactions [26], [67], [68], the initial selective advantage during its *de-novo* emergence remains enigmatic in the light of the otherwise highly stable, streamlined genomes of Hepadnaviridae.

## Materials and Methods

### Presence/Absence Analyses

Subsequent to our initial tBLASTx searches [69] (cutoff e-value 1e–10) for sequence similarity between DHBV/eZHBV_C and non-avian amniote genomes, we extracted all resultant BLAST hits (including >5 kb of sequence per eHBV flank) for eTHBV, eCRHBV1, eCRHBV2, eSNHBV1, and eSNHBV2 from turtle, snake, and crocodilian genomes. In the case of genomes that did not yield a tBLASTx hit, we obtained orthologous sequences via BLASTn searches using the aforementioned eHBV flanks. The sequences of each eHBV locus were aligned using MAFFT (E-INS-i, version 6, http://mafft.cbrc.jp/alignment/server/index.html) [70], followed by manual realignment (see S1 Dataset for full sequence alignments). Presence/absence states were ascertained by standards similar to those used for presence/absence of retroposon insertions [71]. Consequently, the shared presence (orthology) of an eHBV is indicated by identity regarding its truncation, orientation, and genomic target site. eHBV absence corresponds to orthologous sequences flanking an empty eHBV target site.

### *In Vitro* Analyses

To complete our turtle and crocodilian sampling, we sequenced orthologous fragments of the eTHBV locus in pleurodiran turtles (*Mesoclemmys dahli*, *Podocnemis expansa*) and the eCRHBV2 locus in the dwarf crocodile (*Osteolaemus* sp.) using standard methods [71]. Briefly, PCR reactions (5 min at 94°C followed by 35–40 cycles of 30 s at 94°C, 30 s at 45–53°C and 45–60 s at 72°C; final elongation of 10 min at 72°C) were performed using specific oligonucleotide primers (see S4 Table), followed by direct sequencing. The sequences were deposited in the European Nucleotide Archive (accession numbers LK391754-LK391756).

### Tests for Non-neutral Evolution

We tested for evidence of non-neutral evolution in eHBV sequences by comparing nested codon models where ω was fixed to 1 or allowed to vary freely in codeml using model 0 on each pair of closely related host species with codon frequency F3X4 [72]. Model fit was assessed using the likelihood ratio test and evidence for non-neutral evolution was defined as rejection of the null model (ω = 1). After removal of premature stop codons and frameshifting indels, we analyzed the non-overlapping and overlapping parts of each ORF separately as coding sites are synonymous in one frame but non-synonymous in others in overlapping ORFs. This in principle allows us to interpret the results of the codon model for the non-overlapping sequences.

### Distances between Orthologous eHBVs and Neutral Substitution Rates

As three of the five non-avian eHBVs are present in orthologous positions in two or more host species, respectively, we estimated nucleotide distances between orthologous sets of sequences. The best-fit model of nucleotide substitution was chosen using jModeltest 2 [73] under the Akaike Information Criterion (HKY model: -lnL 2610.28570) and sequences were analyzed in BASEML [72] using the HKY model under a global clock and considering the respective species tree topologies of Fig. 1A. The calculated node ages (i.e., half of the distance between a given pair of sequences that diverged since the root of the species tree) were 0.027 for eCRHBV1 (2,501 bp), 0.008 for eCRHBV2 (1,650 bp), and 0.151 for eTHBV (910 bp). In order to subsequently date eHBV divergences using these distances, we used neutral substitution rates reported by Green et al. [30]. For crocodilians, they estimated a neutral rate of 3.9×10^−10^ substitutions/site/year based on a whole-genome alignment between saltwater crocodile and American alligator. In the case of turtles, we used neutral substitution rates based on conserved 4-fold degenerate sites [30], namely 8.43×10^−10^ substitutions/site/year for *Pelodiscus* sp. and 4.77×10^−10^ substitutions/site/year for *Chelonia mydas*.

### HBV Genome Annotation

We aligned nucleotide sequences of eTHBV, eSNHBV1, eSNHBV2, eCRHBV1, and eCRHBV2 to the whole genomes of DHBV and eZHBV_C [10]. The resulting alignment was used to localize putative start and stop codon positions for hepadnaviral ORFs, as well as to identify frameshifts. Nucleotide and amino acid sequences of hepadnaviral protein-coding genes were reconstructed after replacement of premature stop codons with “NNN” in the nucleotide sequences and removal of frameshift mutations (see S2 Dataset for the near-complete genome of the crocodilian eCRHBV1). Nucleotides of frameshifting insertions were omitted and frameshifting deletions were accounted for by insertion of “N” residues.

DR sequences were identified in the near-complete eCRHBV1 genome by screening the region around the *pol* ORF end and the *preC/C* ORF start for identical direct repeat sequences. Furthermore, we analyzed the sequence of the aforementioned region in mfold [74] to locate and reconstruct the putative ε hairpin structure.

### Phylogenetic Analyses

We aligned polymerase protein sequences from 47 orthohepadnaviruses, 84 avihepadnaviruses, 3 full-length avian eHBVs (eZHBV_C [10], eBHBV1+eBHBV2 [19]), as well as the crocodilian eCRHBVs (and, for S4A–B Figure, also the snake eSNHBVs) using MAFFT and then manually realigned these. Some N-terminal sites of the alignments were problematic (i.e., the spacer region of the Pol protein) and were thus excluded from further analyses. Note that concerning avian eHBVs, we only considered full-length EVEs (eBHBV1+eBHBV2 from budgerigar [18], [19] and eZHBV_C from Neoaves [10]) to minimize missing data in our analyses. Non-hepadnaviral outgroups comprise reverse transcriptase sequences from representatives of caulimoviruses, retroviruses, and retrotransposons, all of which were manually aligned to the aforementioned HBV alignment. C-terminal and N-terminal residues were removed from outgroup sequences if they could not be aligned to the HBV Pol protein. For generating the precore/core protein sequence alignment, the same ingroup sampling was used as for the Pol protein, in addition to the turtle eTHBV fragment (and, for S4C Figure, also the snake eSNHBV2 fragment) that spans most of the *preC/C* ORF. After processing with MAFFT and manual realignment, capsid protein sequences from representatives of retroviruses and retrotransposons were added and manually aligned while strictly following the helix structure-based alignment of ref. [75].

Maximum likelihood phylogenetic analyses of the final Pol and PreC/C alignments (see S3 Dataset and S4 Dataset, respectively) were carried out using RAxML [76] (version 7.4.7). Amino acid substitution models were chosen based on model testing in MEGA5 [77] using default parameters with either all alignment sites or after partial deletion of missing data (95% cutoff for site coverage). The respective best-fit models were chosen for Pol (JTT+G and rtREV+G) and PreC/C (JTT+G and WAG+G) and, as they resulted in the same topologies with similar bootstrap support, we included only the results using the model tested with all alignment sites (JTT+G for both Pol and PreC/C) in Fig. 3 and S4 Figure.

### GC Window Analyses

GC content for windows of X nucleotides length was determined by tallying up all A, C, T and G nucleotides. Only windows where the number of gaps or ambiguous nucleotides was smaller than half of the length of the window were considered.

### Overprinting Analyses

To compare similarity in codon usage between the putatively overprinted and the non-overlapping regions of the genome and older overlapping reading frames, the number of occurrences for each codon in the sequence was tallied. Spearman's rho was then used to obtain correlation coefficients between sequences as a measure of similarity. Codons for which the number of occurrences of the amino acid did not exceed its degeneracy were filtered out. This approach is similar to the method of Pavesi et al. [60], and assumes that in the case of overlapping reading frames a more newly arisen overprinted sequence will initially be less similar in terms of codon usage to the remainder of the genome because its reading frame is shifted. To assess whether our *X* sequence was sufficiently long to infer overprinting from this test, we performed a subsampling analysis. Here, we asked how often a randomly selected fragment from the old overlapping reading frame of the same length as the *X* gene gave a correlation with the non-overlapping region that was as weak as or weaker than the value obtained for *X* (1000 bootstraps). Note that this codon similarity analysis assumes that usage ought to be fairly uniform across all open reading frames in the viral genome.

## Supporting Information

S1 Figure GC content of non-avian eHBV insertion loci. (A) eTHBV locus. (B) eCRHBV1 locus. (C) eCRHBV2 locus. GC content of eHBVs (grey background) and their flanking sequences (white background) was analyzed using 100-bp windows.
(TIF)

S2 Figure Alignment of hepadnaviral ε sequences. Apart from the priming bulge (boxed), there is little sequence similarity between ε sequences of avian eHBVs+HBVs, mammalian HBVs, and the crocodilian eCRHBV1.
(TIF)

S3 Figure Evolution of hepadnaviral GC content. (A) GC content of *Orthohepadnavirus* genomes using 200-bp windows. (B) GC content of *Avihepadnavirus*+eHBV genomes in 200-bp windows. (C) Reconstruction of hepadnaviral whole-genome GC content across the Pol protein phylogeny excluding outgroups. (D) GC content of RNase H domains in the presence of overlap with *X* in mammalian HBVs and in the absence of *X* in other HBV and in an outgroup (Caulimoviridae).
(TIF)

S4 Figure Phylogeny of Hepadnaviridae including the short fragments of snake eHBVs. RAxML analyses were conducted using the same alignments and parameters as in Fig. 3B–C with the addition of eSNHBVs. (A) Pol tree of eSNHBV1. (B) Pol tree of eSNHBV2. (C) PreC tree of eSNHBV2. As the eSNHBV1 and eSNHBV2 fragments do not overlap, we analyzed their Pol sequences separately. Only ML bootstrap values ≥50% are shown.
(TIF)

S5 Figure *X* gene subsampling analyses. Distribution of randomized rho values based on 1000 samples from *preS* (overlapping) of the length of the *X* (overlapping) ORF. The observed correlation between the codon frequencies of *X* and non-overlapping regions is marked by the red line. The black lines denote the 5% and 95% percentiles of the simulated distribution.
(TIF)

S1 Table Insertion/deletion frameshifts and premature stop codons in non-avian eHBVs.
(XLS)

S2 Table Summary of tests for non-neutral evolution.
(XLS)

S3 Table Summary of overprinting analyses.
(XLS)

S4 Table Oligonucleotide primer sequences.
(XLS)

S1 Dataset Full sequence alignments of the five non-avian eHBV insertion loci.
(TXT)

S2 Dataset Reconstructed gene and protein sequences of a near-complete crocodilian eHBV genome.
(TXT)

S3 Dataset Pol sequence alignment used for phylogenetic analyses of Fig. 3B.
(TXT)

S4 Dataset PreC/C sequence alignment used for phylogenetic analyses of Fig. 3C.
(TXT)
