# Supplementary figures and images for: Early Mesozoic Coexistence of Amniotes and Hepadnaviridae
Source: PLoS Genet. 2014 Dec 11;10(12):e1004559. doi: 10.1371/journal.pgen.1004559 (PMC4263362; doi:10.1371/journal.pgen.1004559)

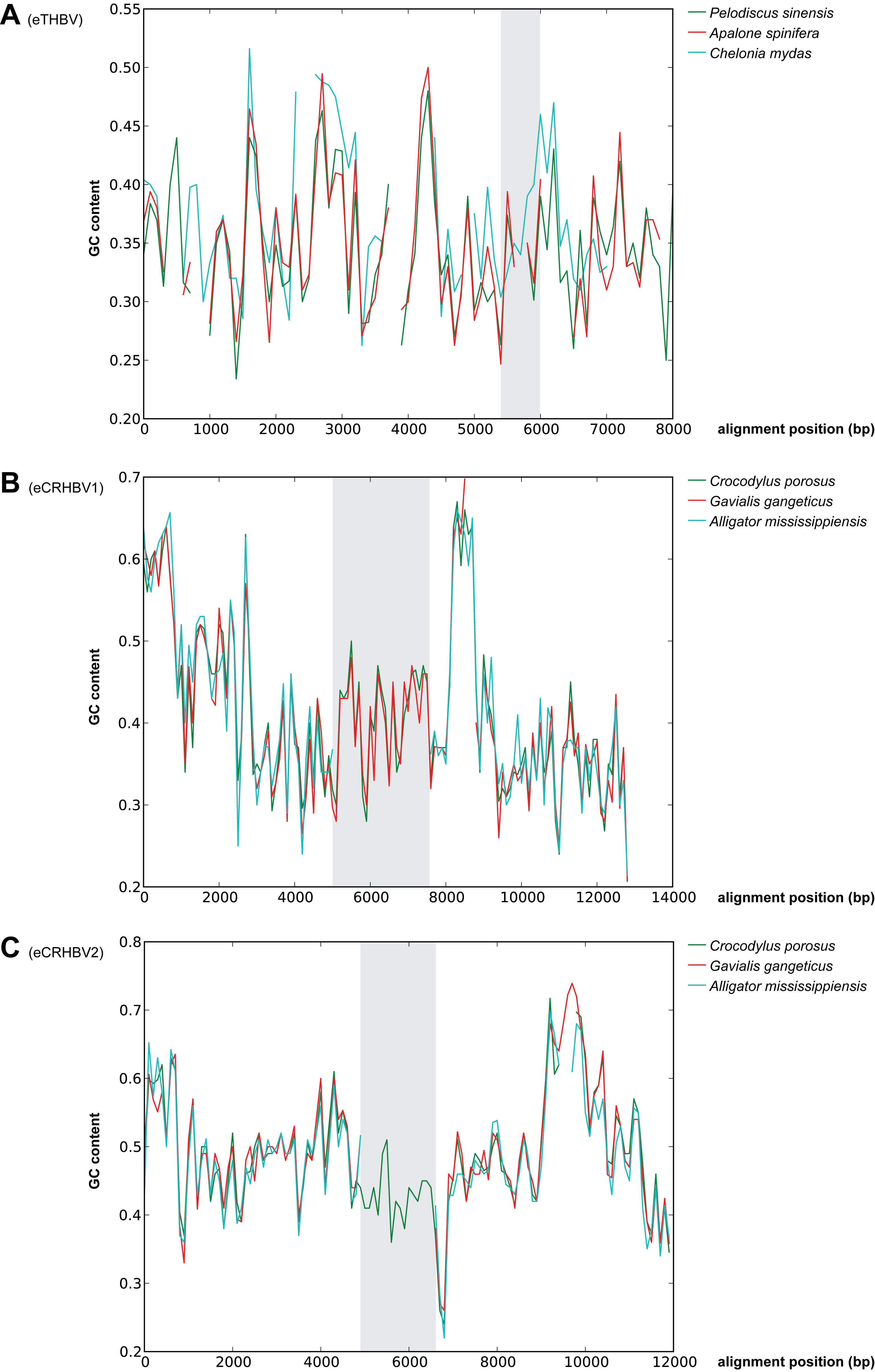

Supplement: S1 Figure — GC content of non-avian eHBV insertion loci. (A) eTHBV locus. (B) eCRHBV1 locus. (C) eCRHBV2 locus. GC content of eHBVs (grey background) and their flanking sequences (white background) was analyzed using 100-bp windows. (TIF) [file pgen.1004559.s001.tif]

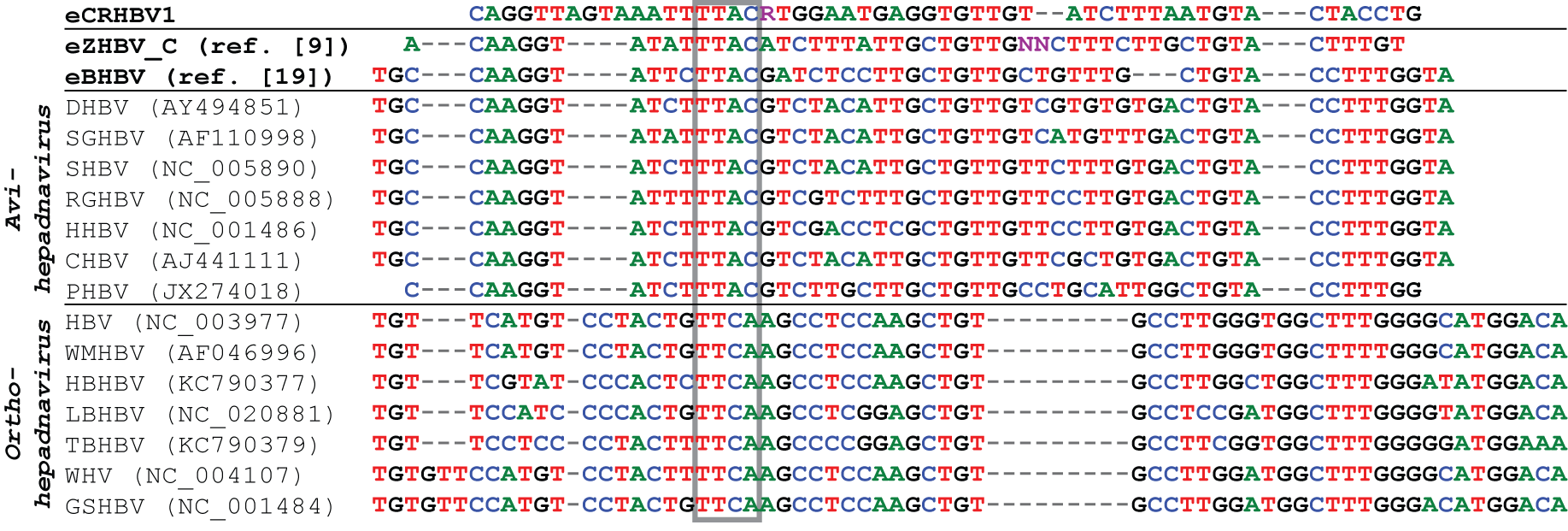

Supplement: S2 Figure — Alignment of hepadnaviral ε sequences. Apart from the priming bulge (boxed), there is little sequence similarity between ε sequences of avian eHBVs+HBVs, mammalian HBVs, and the crocodilian eCRHBV1. (TIF) [file pgen.1004559.s002.tif]

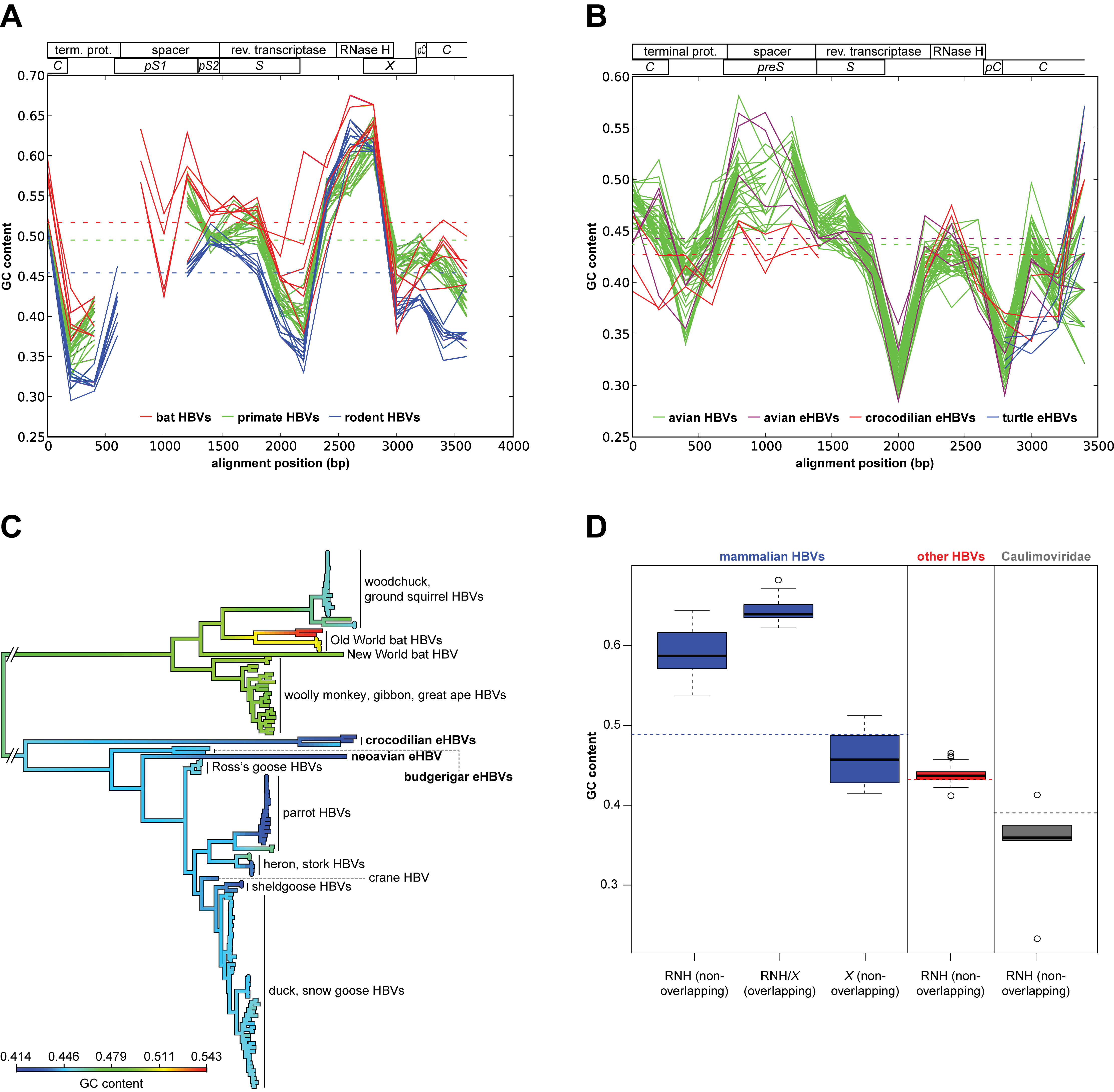

Supplement: S3 Figure — Evolution of hepadnaviral GC content. (A) GC content of Orthohepadnavirus genomes using 200-bp windows. (B) GC content of Avihepadnavirus+eHBV genomes in 200-bp windows. (C) Reconstruction of hepadnaviral whole-genome GC content across the Pol protein phylogeny excluding outgroups. (D) GC content of RNase H domains in the presence of overlap with X in mammalian HBVs and in the absence of X in other HBV and in an outgroup (Caulimoviridae). (TIF) [file pgen.1004559.s003.tif]

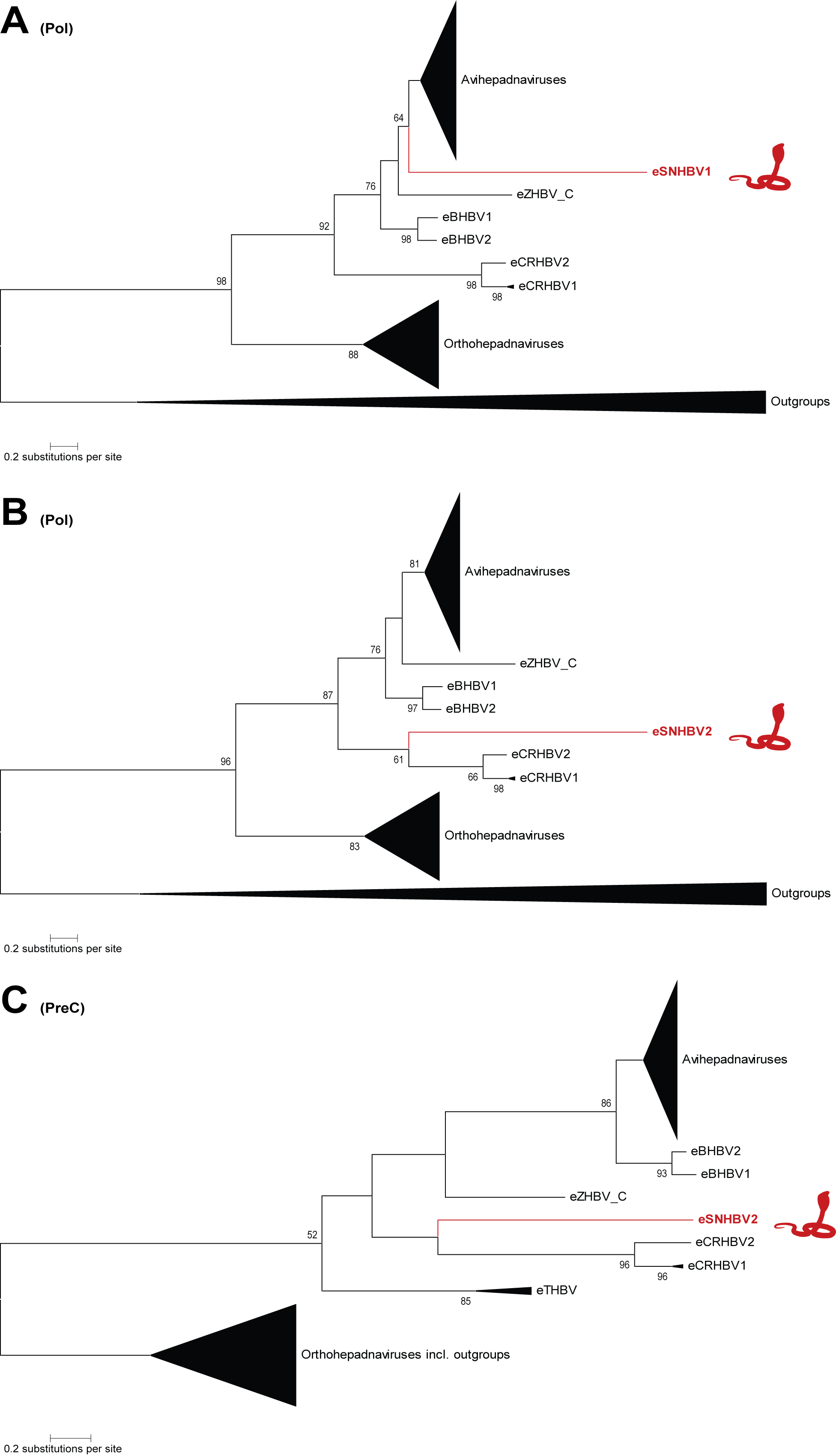

Supplement: S4 Figure — Phylogeny of Hepadnaviridae including the short fragments of snake eHBVs. RAxML analyses were conducted using the same alignments and parameters as in Fig. 3B–C with the addition of eSNHBVs. (A) Pol tree of eSNHBV1. (B) Pol tree of eSNHBV2. (C) PreC tree of eSNHBV2. As the eSNHBV1 and eSNHBV2 fragments do not overlap, we analyzed their Pol sequences separately. Only ML bootstrap values ≥50% are shown. (TIF) [file pgen.1004559.s004.tif]

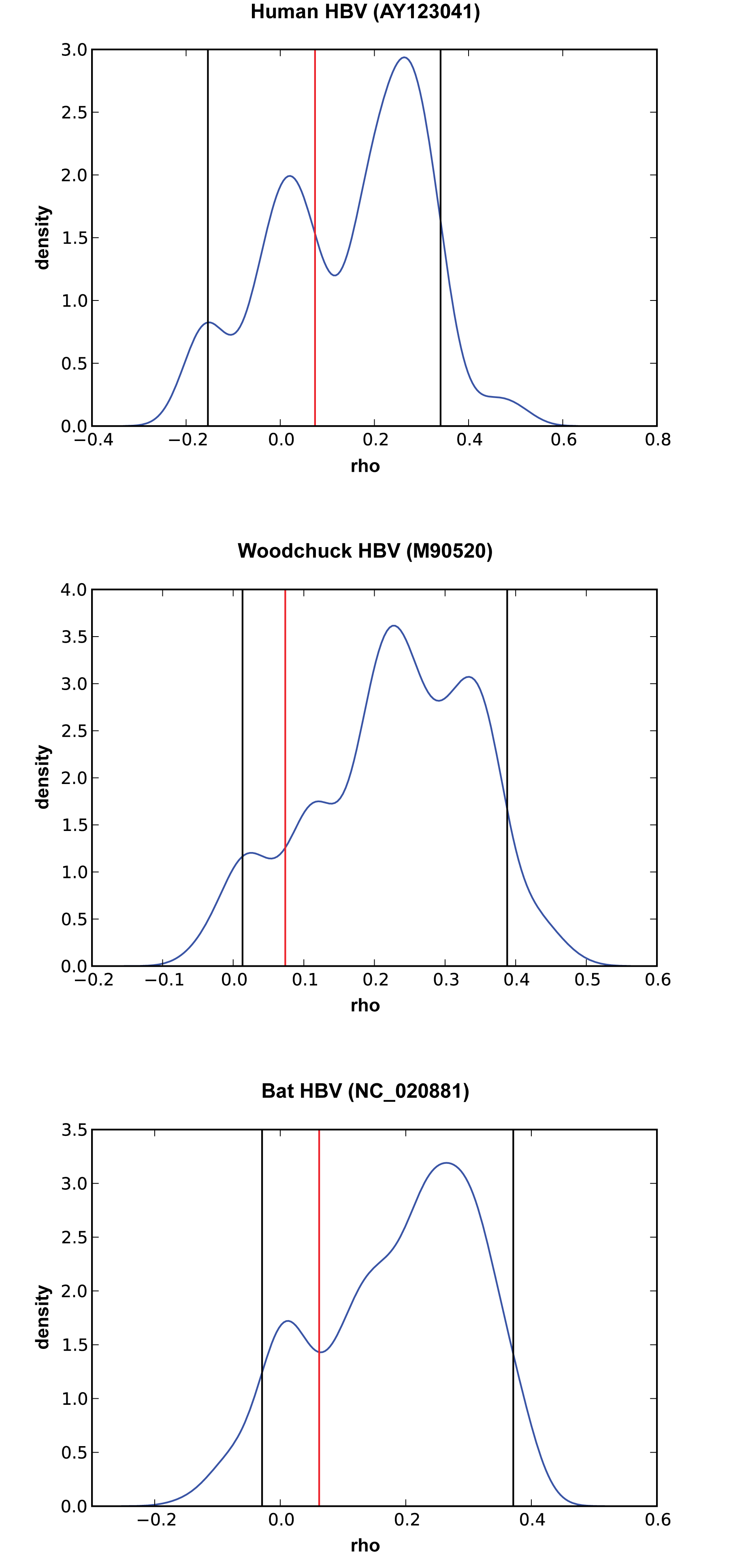

Supplement: S5 Figure — X gene subsampling analyses. Distribution of randomized rho values based on 1000 samples from preS (overlapping) of the length of the X (overlapping) ORF. The observed correlation between the codon frequencies of X and non-overlapping regions is marked by the red line. The black lines denote the 5% and 95% percentiles of the simulated distribution. (TIF) [file pgen.1004559.s005.tif]
